# Foot posture index and body composition measures in children with and without developmental coordination disorder

**DOI:** 10.1371/journal.pone.0265280

**Published:** 2022-03-14

**Authors:** Timothy Tsz Ting Yam, Shirley Siu Ming Fong, William Wai Nam Tsang

**Affiliations:** 1 Department of Physiotherapy, School of Nursing and Health Studies, Hong Kong Metropolitan University, Hong Kong, Hong Kong; 2 School of Public Health, LKS Faculty of Medicine, The University of Hong Kong, Hong Kong, Hong Kong; 3 Department of Health and Physical Education, The Education University of Hong Kong, Hong Kong, Hong Kong; Universiti Sains Malaysia, MALAYSIA

## Abstract

**Background:**

Foot posture which forms the distal supporting structure influences on postural stability. Children with developmental coordination disorder (DCD) who are more likely to be overweight or obese may present with flat foot with symptoms that affect daily activities. The aim of this study was to compare the foot posture and body composition measures between children with and without DCD. In addition, this study aimed to investigate the relationship between foot posture and fat percentage.

**Methods:**

Fifty-nine children with DCD (mean age = 8.07±1.10) and sixty-two typically developing children (mean age = 7.97±1.05) were recruited to the DCD and control group respectively. All children received a foot posture assessment and a whole-body dual-energy X-ray absorptiometry (DXA) scan. Foot Posture Index 6 (FPI-6) total scores, sub-scores and lower limb body composition measures including fat mass, lean mass, total mass, fat percentage and fat mass index were measured.

**Results:**

Children with DCD revealed a significantly higher FPI-6 left (1.12; 95% CI: 0.172, 2.061) and right (1.15; 95% CI: 0.218, 2.079) total score. FPI-6 sub-scores (talar head palpation and abduction/adduction forefoot on rearfoot) illustrated significant differences between children with and without DCD. Children with DCD had a significantly higher total fat mass (1247.48g; 95% CI: 121.654, 2373.304), total fat percentage (1.82%; 95% CI: 0.115, 3.525) and fat mass index (0.56kg/m^2^; 95% CI: 0.036, 1.069). There was a significant relationship between FPI-6 right total score and total fat percentage.

**Conclusion:**

The findings of this study showed that children with DCD exhibited significantly more pronated foot posture and higher body composition measures compared to typically developing children. Moreover, with FPI-6 right total score significantly related to the total fat percentage, it may require more than just detecting abnormal foot structures in children with DCD but also promoting a healthy lifestyle to prevent obesity.

## Background

Developmental coordination disorder (DCD) is a neurodevelopmental condition which greatly affects normal activities of daily living. DCD constitutes around 6% of the school-aged population [1]. Previous studies have illustrated less competent gait and balance performances in children with DCD [2, 3]. This may be due to physiological and psychosocial factors which they exhibit greater gait parameter variability and a generally lower self-confidence in physical and functional domains.

Foot posture (neutral, pronated or supinated) is crucial to providing a solid base of support to maintain static and dynamic balance stability [4]. The shape of the sole directly influences the ground reaction force received by the foot [5]. The arches of our foot are mainly formed by the tarsal and metatarsal bones reinforced with numerous ligaments and tendons [6]. This ultimately serves to support the body weight during locomotion and other physical activities. Children typically develop an adult-like medial longitudinal arch (MLA) at the age of 4–5 years old [7].

Flat foot (pes planus) is a condition in which there is a rearfoot eversion with or without lowering of the foot arch. This may progress to symptomatic flat foot, of which daily activity functions are affected. Flat foot is particularly common in school-aged children with a prevalence rate of up to 59% [8]. This prevalence rate is even higher in children who are obese/overweight and/or with developmental delay [9–11]. The development of the foot’s posture, especially that of the MLA, which is crucial to the dissipation of ground reaction force, may be influenced by several factors including the amount of physical activity, body weight and the fatigue rate of the intrinsic foot muscles [12–14].

Obese and overweight children are twice as likely to have flat foot when compared to children with normal weight which may be a compensatory mechanism to build a thicker fat pad at the midfoot to counter the excessive body weight [15, 16]. It has been suggested that flat foot is associated with the body composition [17, 18]. More specifically the amount of muscle mass (appendicular lean mass index) [3] may be associated with the developmental of flat foot. Flat foot in children may interfere with their activities of daily living and quality of life [19]. It is imperative to investigate the prevalence of flat foot among children with DCD and its relationship to body composition measures.

The aim of this cross-sectional study was i) to compare the Foot Posture Index (6-item) (FPI-6) and body composition (fat percentage and fat mass) between children with and without DCD; and ii) to investigate the associations of FPI-6 scores and fat mass among children with DCD. We hypothesized that a more pronated foot posture would be illustrated in children with DCD than in typically developing children and a significant association between the FPI-6 total scores and fat content in children with DCD.

## Methods

### Participants

Children aged 6 to 9 years were recruited from Hong Kong local primary schools from May to August 2016. A total of one-hundred and twenty-one children met the criteria to participate in this study. Fifty-nine were allocated to the DCD group and sixty-two to the control group.

The two-step method was used to determine children with DCD [3]: i) select children aged 6–9 years with fine and gross motor problems that affect daily activities (DCD questionnaire (DCDQ) was used to provide additional information on motor deficits) [20]; and ii) screen and assess children using the Movement Assessment Battery for Children, 2^nd^ edition [21] and the Diagnostic and Statistical Manual of Mental Disorders 5^th^ Edition (DSM-V) [1]. A MABC-2 score ≤ 15^th^ percentile indicates motor skills are below that of the expected age. Exclusion criteria included i) history of serious lower limb injuries (e.g., fractures); ii) excessive disruptive behaviour; iii) inability to follow instructions; iv) metal implants; and v) disorders which may influence the ability to maintain static posture for prolonged periods.

Ethical approval was obtained from the Human Research Ethics Committee of the University of Hong Kong and the study was conducted in the accordance with the Declaration of Helsinki (2013). Written informed consent was obtained from the children and their parents. All experimental procedures were conducted by two physiotherapists who are licensed to operate the dual-energy X-ray absorptiometry (DXA) machine.

### Outcome measurements

#### Demographics

Children and their parents provided the demographic information (age, sex, height, body weight, leg length) and medical history (history of lower limb injuries, comorbid conditions and disorders). Body weight was measured in kilograms using an electronic scale (A and D, UC-231, Tokyo, Japan) and height was measured in centimeters with a stadiometer (seca 213, Seca, CA, USA).

#### Foot Posture Index 6 (FPI-6)

The foot posture of each child was evaluated once using the FPI-6 by the same physiotherapist since it has good intra-observer reliability [22]. Children stood in a relaxed-stance position with double limb support and arms at their sides. They were instructed to stand still and look straight ahead for around 2 minutes. The FPI-6 is a valid clinical assessment on the foot posture with excellent inter-rater reliability and moderate diagnostic accuracy to determine paediatric flexible flat foot [23]. The six sub-scores include: i) talar head palpation; ii) curves above and below the lateral malleolus; iii) inversion/eversion of the calcaneus; iv) prominence in the region of the talo-navicular joint (TNJ); v) congruence of the medial longitudinal arch; and vi) abduction/adduction of forefoot on rearfoot [24]. Each item is given a score between -2 and +2 adding up to a total score of -12 to +12. Children were categorized into five representing groups based on their final score: i) highly pronated (+10 to +12); ii) pronated (+6 to +9); iii) normal (0 to +5); iv) supinated (-1 to -4); and v) highly supinated (-5 to -12).

#### DXA-derived body compositions

A single whole-body DXA scan (Horizon A, Hologic Inc., Bedford, MA) was administered to each child. The children were instructed to wear loose clothing free of metal or plastic parts. As illustrated in the DXA manual [25], children were positioned in supine with palm facing down vertically at the side of the body. The big toes were in contact by internally rotating the hips. The children were told to remain still and breathe normally for the whole duration of the scan.

### Statistical analyses

The sample size was calculated using the G*Power version 3.1.0. software (Franz Faul, Universitat Kiel, Germany). A previous study investigated the foot posture of children with and without patellofemoral pain syndrome with effect sizes ranging from 0.47 to 0.65 [26]. An effect size of 0.50 was used in this study to calculate the sample size with a statistical power at 80% and an alpha level of 5% (two-tailed). A minimum sample size of 51 children per group was required to detect a between-group difference.

Statistical analyses were performed using the Statistical Package for Social Science (SPSS) 23.0 software (IBM, Armonk, NY). The normality criterion was reassured with the Shapiro-Wilk test. Demographics information was compared using the independent t-test (continuous data) and the chi-square test (categorical data). The multivariate analysis of variance (MANOVA) was used to detect between-group differences in the DXA body composition outcomes and FPI-6 scores. To explore the relationship between DXA outcomes and FPI-6 scores, Pearson’s correlation coefficients (r) were used.

## Results

### Demographics

Table 1 illustrates the demographic characteristics of the children. There were no significant differences in age, gender, height, body weight and body mass index (BMI) between the DCD and control group. Unsurprisingly, the Movement Assessment Battery for Children, 2^nd^ edition percentile (MABC-2 percentile) score and DCD total score were significantly different between the two groups (p < 0.001).

**Table 1:** Characteristics of the participants.

	DCD (n = 59)	Control (n = 62)	p value
Age (years)	8.07 ± 1.10	7.97 ± 1.05	0.651
Sex			0.228
Male (n, %)	45 (76.3)	39 (65.0)	
Female (n, %)	14 (23.7)	23 (35.0)	
Height (cm)	126.91 ± 9.81	125.44 ± 8.19	0.562
Body weight (kg)	26.40 ± 7.31	24.72 ± 4.52	0.231
Body mass index (kg/m^2^)	16.13 ± 2.76	15.60 ± 1.37	0.284
Leg length (cm)	64.16 ± 6.82	64.98 ± 5.06	0.548
DCD questionnaire 2007 total score	43.14 ± 12.35	57.06 ± 11.66	**<0.001** ^**a**^
MABC-2 (percentile)	7.99 ± 5.76	51.06 ± 22.69	**<0.001** ^**a**^
Comorbid conditions			
Attention deficit hyperactivity disorder (n, %)	4 (6.8)	7 (11.3)	
Autism spectrum disorder (n, %)	10 (16.9)	3 (4.8)	
Foot Posture Index—6 Score			
Left total score	6.41 ± 3.04	5.29 ± 2.16	**0.021** ^**a**^
Right total score	6.41 ± 2.95	5.26 ± 2.17	**0.016** ^**a**^

Means ± standard deviations are presented unless otherwise specified.

^a^Significant difference at p < 0.05

DCD: developmental coordination disorder; MABC-2: Movement Assessment Battery for Children 2^nd^ edition

### FPI-6 total scores

The FPI-6 sub- and total scores between the two groups are illustrated in Table 2. Results revealed a significantly higher FPI-6 total score in the DCD group (Left total score: F_1,118_ = 7.211, p = 0.021; Right total score: F_1,118_ = 5.901, p = 0.016). For both sides of FPI-6 sub-scores, talar head palpation (Left: F_1,118_ = 0.065, p = 0.044; Right: F_1,118_ = 2.755, p = 0.040) and abduction/adduction of forefoot on rearfoot (Left: F_1,118_ = 20.347, p = 0.018; Right: F_1,118_ = 24.107, p = 0.010) scores were significantly higher in the DCD group. Additionally, the prominence in the region of the TNJ score was significantly higher in the DCD group for the left side (F_1,118_ = 20.347, p = 0.018).

**Table 2:** Comparison of outcome measures between the DCD group and control group.

	DCD (n = 59)	Control (n = 62)	Mean Difference^b^	95% Confidence Interval	F_1,118_	P value
**Foot Posture Index– 6**						
Left—Rearfoot						
Talar head palpation	0.95 ± 0.66	0.73 ± 0.55	0.22	0.006, 0.440	0.065	**0.044** ^**a**^
Curves above and below the lateral malleolus	1.24 ± 0.84	1.08 ± 0.66	0.16	-0.114, 0.427	13.532	0.254
Inversion/eversion of the calcaneus	1.44 ± 0.68	1.34 ± 0.63	0.10	-0.132, 0.336	1.013	0.391
Left–Forefoot						
Prominence in the region of the TNJ	1.24 ± 0.68	0.87 ± 0.74	0.37	0.111, 0.621	0.085	**0.005** ^**a**^
Congruence of the medial longitudinal arch	1.12 ± 0.67	1.06 ± 0.60	0.06	-0.174, 0.283	2.077	0.640
Abd/adduction of forefoot on rearfoot	0.42 ± 0.56	0.21 ± 0.41	0.21	0.037, 0.391	20.347	**0.018** ^**a**^
Left Total	6.41 ± 3.30	5.29 ± 2.16	1.12	0.172, 2.061	7.211	**0.021** ^**a**^
Right–Rearfoot						
Talar head palpation	0.98 ± 0.60	0.76 ± 0.59	0.22	0.010, 0.440	2.755	**0.040** ^**a**^
Curves above and below the lateral malleolus	1.29 ± 0.77	1.06 ± 0.67	0.23	-0.036, 0.483	6.540	0.091
Inversion/eversion of the calcaneus	1.42 ± 0.68	1.24 ± 0.67	0.18	-0.060, 0.424	0.534	0.140
Right–Forefoot						
Prominence in the region of the TNJ	1.15 ± 0.69	0.97 ± 0.79	0.18	-0.083, 0.452	0.932	0.174
Congruence of the medial longitudinal arch	1.14 ± 0.71	1.03 ± 0.60	0.11	-0.132, 0.339	5.001	0.387
Abd/adduction forefoot on rearfoot	0.42 ± 0.56	0.19 ± 0.40	0.23	0.055, 0.405	24.107	**0.010** ^**a**^
Right Total	6.41 ± 2.95	5.26 ± 2.17	1.15	0.218, 2.079	5.901	**0.016** ^**a**^
**DXA outcomes**						
Left leg fat mass (g)	1735.27 ± 852.71	1584.74 ± 473.03	150.53	-96.124, 397.183	22.139	0.229
Left leg lean mass (g)	2526.81 ± 815.61	2538.29 ± 569.10	-11.48	-263.635, 240.671	8.450	0.928
Left leg total mass (g)	4389.98 ± 1623.28	4257.37 ± 993.97	132.61	-349.309, 614.533	15.298	0.587
Left leg fat percentage (%)	37.83 ± 7.34	36.94 ± 4.50	0.89	-1.281, 3.078	18.434	0.416
Right leg fat mass (g)	1797.90 ± 878.27	1642.18 ± 462.55	155.72	-95.242, 406.684	24.679	0.222
Right leg lean mass (g)	2586.51 ± 855.78	2610.97 ± 591.02	-24.46	-288.115, 239.203	7.164	0.855
Right leg total mass (g)	4515.88 ± 1682.67	4390.19 ± 1004.21	125.69	-370.314, 621.690	15.297	0.617
Right leg fat percentage (%)	38.23 ± 7.25	37.21 ± 4.26	1.02	-1.113, 3.145	16.333	0.347
Total fat mass (g)	8979.90 ± 7695.12	7732.42 ± 1988.65	1247.48	121.654, 2373.304	25.019	**0.030** ^**a**^
Total lean mass (g)	16991.29 ± 3661.89	16296.69 ± 3387.90	694.60	-574.528, 1963.734	2.225	0.281
Total mass (g)	26817.49 ± 7389.05	25127.90 ± 4625.67	1689.59	-518.061, 3897.238	14.327	0.132
Total fat percentage (%)	32.34 ± 5.89	30.52 ± 3.28	1.82	0.115, 3.525	25.806	**0.037** ^**a**^
Fat mass/Height^2^ (kg/m^2^)	5.39 ± 1.86	4.83 ± 0.85	0.56	0.036, 1.069	26.817	**0.036** ^**a**^

Means ± standard deviations are presented unless otherwise specified.

^a^Significant difference at p < 0.05

^b^: Mean difference = DCD–control

DCD: developmental coordination disorder; TNJ: talo-navicular joint; DXA: dual-energy X-ray absorptiometry

### DXA body compositions

Table 2 illustrates the DXA body composition outcomes which include lower limb fat mass and lean mass. The DCD group had a significantly higher total fat mass and total fat percentage (F_1,118_ = 25.019, p = 0.030 and F_1,118_ = 25.806, p = 0.037 respectively). A similar pattern was observed when taking height into account (fat mass index) (F_1,118_ = 26.817, p = 0.036). However, no between-group differences were detected when investigating the limbs separately (p > 0.05).

### Relationship between FPI-6 scores and DXA body compositions

Pearson’s correlation analysis revealed a significant relationship between FPI-6 right total score and total fat percentage (r = -0.281, F_1,118_ = 4.897, p = 0.031). Further linear regression showed that total fat percentage (t = -2.213, p = 0.031) was a significant predictor of FPI-6 right total score and accounted for 7.9% of the variance (adjusted 6.3%) (Table 3).

**Table 3:** Relationship between FPI-6 scores and DXA outcomes in children with DCD.

	Left Rearfoot 1		Left Forefoot 1		Left Forefoot 3		Left total		Right Rearfoot 1		Right Forefoot 3		Right total	
	r	p	r	p	r	p	r	p	r	p	r	p	r	p
Total fat mass	-0.204	0.121	-0.170	0.197	0.126	0.341	-0.095	0.473	-0.215	0.103	-0.127	0.338	-0.182	0.167
Total fat percentage	-0.101	0.447	-0.194	0.140	-0.070	0.600	-0.145	0.274	-0.143	0.280	-0.246	0.060	**-0.281**	**0.031** ^**a**^
Fat mass/Height^2^ (kg/m^2^)	-0.168	0.204	-0.140	0.291	0.069	0.605	-0.090	0.499	-0.192	0.145	-0.136	0.304	-0.168	0.203

^a^Significant difference at p < 0.05

FPI-6: foot posture index 6; DXA: dual-energy X-ray absorptiometry; DCD: developmental coordination disorder

## Discussion

The FPI-6 total scores obtained in our study (DCD: Left = 6.41 ± 3.30, Right = 6.41 ± 2.95; Control: Left = 5.29 ± 2.16, Right = 5.26 ± 2.17) were comparable and in agreement with previous studies where FPI-6 scores in children range from a mean score of 4–6 points (age 6 years) to 2–3 points (age 10–14 years) [27, 28]. The results revealed that children with DCD exhibited a 1.12 to 1.15 higher FPI-6 total score than the control group. This suggests that children with DCD are more likely to have flat foot [13] which may be the reason why children with DCD exhibit balance and gait deficits [2, 3]. When inspecting the FPI-6 sub-scores, i) talar head palpation; ii) abduction/adduction of forefoot on rearfoot; and iii) prominence in the region of the TNJ were more evident and palpable in the DCD group.

The talus, navicular and TNJ are essential body-weight-supporting foot structures which are involved in the development of flat foot [29–32]. With navicular as the keystone forming the MLA, the interaction and dynamics between talus and navicular is crucial [30]. Our results illustrate a prominence in the TNJ region sub-score on the left side with a less palpable talus on the medial aspect in children with DCD. Quantifying the navicular height is a valid and reliable way to assess static foot posture in children [31]. This may be the compensatory outcome of flat foot where the talar neck is more aligned with the sagittal foot axis, causing the collapse of the longitudinal arches [31]. Aside from the talar position, other factors such as physical activity level and body weight may be attributable to the development of flat foot [9, 16, 33].

Children with DCD often demonstrate poorer motor proficiency when compared with typically developing children. This is related to their higher body weight, higher body mass index and compromised cardiorespiratory fitness [34]. Children who are obese are two to three times more likely to develop flat foot with a pronated heel and decreased ankle dorsiflexion [11, 33]. However, studies have revealed that anthropometric measures are not indicatives of the paediatric foot posture where a higher BMI does not necessarily reflect a more pronated foot type in children [26, 35]. Thus, BMI might not be an ideal and a representable measurement when investigating the relationship between body composition and foot posture. Conversely, an increase in adiposity is associated with a flat foot, reflected by a higher arch index [17]. Furthermore, besides obese children, overweight children already display greater midfoot contact with the ground [36]. This suggests that foot structure is affected as early as preschool years in children who are either overweight or obese. Our results revealed that children with DCD illustrated a higher total fat mass (8979.90g), total fat percentage (32.34%) and fat mass index (5.39 kg/m^2^). The adverse changes of the foot structures will likely exacerbate if the weight condition worsens into adulthood.

Furthermore, the total fat percentage had a correlation with FPI-6 right total score in the DCD group. This agrees with the notion that adipose tissue may be associated to the changes of the foot structure. Although studies have shown that a higher BMI or body weight is not associated with a more pronated foot in children and adolescents [37, 38], excessive amount of body fat percentage significantly lowers the MLA [17]. A thicker fat pad increases the amount of deformable soft tissue forming a larger contact area and a higher pressure on the foot [39–41]. As a higher body weight is speculated to influence lower limb muscle activities [42] and pain in the foot and lower limbs [43], the weight-related flattening of MLA gives further insight on the differential muscle activation patterns in children with DCD during walking [3]. Whether a higher body weight and total fat percentage have comparable effects on plantar pressure and structure remains unknown.

Childhood obesity is a worldwide problem with motor deficits linking to higher BMI. A higher body weight influences the force distribution of the midfoot [36]. This study along with previous studies suggest that body weight and adipose content may also be attributable to the foot’s structure. This ultimately may influence more complex factors such as physical inactivity and sedentary lifestyle [44]. Since childhood obesity is five times more likely to persist into adulthood [45], this may lead to overstress to the musculoskeletal system at a later age. Furthermore, obese children who do not currently have foot structural abnormalities could at a very high risk of developing flat foot without any interventions for weight loss [38]. Thus, aside from detecting abnormal foot structures, it may just be as important to promote a healthy lifestyle to prevent obesity in children with DCD.

To the best of our knowledge, this is the first study to investigate the relationship between body composition measures and flat foot condition in children with DCD. Further research is needed to explore the influence of body composition measures and differentiate the effects of body weight, fat percentage and lean mass on foot structures.

### Limitations

There are some limitations for this study. FPI-6 is a subjective-based assessment of the foot posture. Although having the same physiotherapist perform the foot assessments eliminated inter-operator error, the use of an objective measure such as pressure sensor platforms would provide more insight, with greater accuracy, on the relationship between the foot’s structure and body composition outcomes. Secondly, this study was a cross-sectional study. Thus, it does not provide information on the maturation process of the foot posture. Finally, foot posture assessments were conducted in the static standing position. The results cannot be translated to dynamic movements such as walking. Further research is required to investigate the extent of which flat foot affects locomotion in children with DCD.

## Conclusion

Children with DCD are more likely to develop a more pronated foot posture (flat foot). This suggests that the positions of the talar head and TNJ should be observed closely in this population. Compared to typically developing children, children with DCD have a greater total fat mass, total fat percentage and fat mass index. Additionally, a higher total fat percentage has been revealed to be associated with a more pronated foot posture. Further research is warranted to provide more information on the influence of flat foot on locomotion, and its association with body composition measures.

## Supporting information

S1 Dataset (XLSX)
